# The Regulatory Roles of Cerebellar Glycosphingolipid Microdomains/Lipid Rafts

**DOI:** 10.3390/ijms24065566

**Published:** 2023-03-14

**Authors:** Keisuke Komatsuya, Norihito Kikuchi, Tetsuya Hirabayashi, Kohji Kasahara

**Affiliations:** Laboratory of Biomembrane, Tokyo Metropolitan Institute of Medical Science, Tokyo 156-8506, Japan; komatsuya-ks@igakuken.or.jp (K.K.);

**Keywords:** lipid rafts, gangliosides, GPI-anchored proteins, Src-family kinases, heterotrimeric G proteins

## Abstract

Lipid rafts are dynamic assemblies of glycosphingolipids, sphingomyelin, cholesterol, and specific proteins which are stabilized into platforms involved in the regulation of vital cellular processes. Cerebellar lipid rafts are cell surface ganglioside microdomains for the attachment of GPI-anchored neural adhesion molecules and downstream signaling molecules such as Src-family kinases and heterotrimeric G proteins. In this review, we summarize our recent findings on signaling in ganglioside GD3 rafts of cerebellar granule cells and several findings by other groups on the roles of lipid rafts in the cerebellum. TAG-1, of the contactin group of immunoglobulin superfamily cell adhesion molecules, is a phosphacan receptor. Phosphacan regulates the radial migration signaling of cerebellar granule cells, via Src-family kinase Lyn, by binding to TAG-1 on ganglioside GD3 rafts. Chemokine SDF-1α, which induces the tangential migration of cerebellar granule cells, causes heterotrimeric G protein Goα translocation to GD3 rafts. Furthermore, the functional roles of cerebellar raft-binding proteins including cell adhesion molecule L1, heterotrimeric G protein Gsα, and L-type voltage-dependent calcium channels are discussed.

## 1. Introduction

Glycosphingolipid, one of the glycoconjugates, exists in clusters and forms microdomains containing cholesterol in the cell membrane. This review provides several examples of glycosphingolipid microdomain functions and their molecular mechanism in healthy states and diseases of the cerebellum.

## 2. Lipid Rafts

Glycosphingolipids are found in the outer leaflet of the plasma membrane of all vertebrate cells and are thought to play functional roles in the regulation of cellular proliferation and differentiation [1]. Gangliosides, sialic acid-containing glycosphingolipids, are particularly abundant in the nervous system [2]. The species and amounts of gangliosides undergo profound changes during development, suggesting that they may play fundamental roles in this process. The plasma membrane lipids are not homogeneously distributed and the membranes contain microdomains or compartments. Low-density detergent-resistant membrane (DRM) fractions are isolated from cells by sucrose density gradient centrifugation [3]. The membrane fractions are rich in glycosphingolipids, sphingomyelin, cholesterol, glycosylphosphatidylinositol (GPI)-anchored proteins, and a variety of signaling molecules, such as Src family kinases and the α-subunit of the heterotrimeric G proteins. These observations indicate the possible presence of glycosphingolipid-rich microdomains, referred to as lipid rafts, in cells and their involvement in signal transduction [4]. The presence of lipid rafts in vivo is supported by the results of single-molecule imaging techniques [5].

The basic forces driving the formation of lipid rafts are thought to be lipid interactions. Glycosphingolipids are relatively rich in saturated fatty acyl chains, which allows tight packing and confers the characteristic of a high melting temperature. On the other hand, phospholipids are relatively rich in cis-unsaturated fatty acyl chains (kinked structure), which prevents tight packing and confers the characteristic of a low melting temperature. Lipid rafts are thought to exist as phase-separated domains of glycosphingolipids and cholesterol from phospholipids. Src family kinases and the α-subunit of the heterotrimeric G proteins anchor onto the inner leaflet via N-terminal saturated fatty acyl chains lipid modification, palmitoylation, and myristoylation [6]. The nature of phospholipids occupying the cytoplasmic side of lipid rafts is unknown; however, they probably also carry mainly saturated fatty acid chains to optimize packing. Phospholipids in the raft fraction of rat cerebellar granule cells are mainly dipalmitoylphosphatidylcholine.

## 3. GPI-Anchored Proteins

A variety of cell-surface proteins are anchored in the membrane by GPI. GPI-anchored proteins have various functions, such as enzymes, receptors, and adhesion molecules. GPI-anchored proteins have been implicated in transmembrane signaling, nevertheless, they lack intracellular domains. GPI-anchored proteins are mainly associated with lipid rafts. It is thought that association with Src-family kinases in lipid rafts is important for GPI-anchored proteins in signal transduction.

GPI-anchored proteins usually contain two saturated fatty acyl chains in their phosphatidylinositol moiety. The linkage of GPI-anchored proteins to saturated acyl chains was considered to facilitate targeting to lipid rafts. However, experimental evidence has not been presented. GPI-anchored proteins are synthesized as follows: (i) synthesis of GPI; (ii) attachment of GPI to the protein PGAP2; (iii) lipid remodeling of GPI. GPI contains an unsaturated fatty acyl chain at the sn-2 position, whereas mature GPI-anchored proteins have a saturated fatty acyl chain. The first step of the lipid remodeling is removal of the unsaturated fatty acyl chain at the sn-2 position, generating a lyso-GPI-anchored protein intermediate by GPI-phospholipase A2. The second step of lipid remodeling is reacylation of lyso-GPI-anchored proteins with saturated fatty acid by acyltransferase. Kinoshita and his coworkers have been isolating genes involving biosynthesis of Phosphatidyl lnositol Glycan (PIG) and Post GPI Attachment to Proteins (PGAP) from Chinese hamster ovary cell mutants [7]. PGAP3 is GPI-phospholipase A2. PGAP2 is acyltransferase of sn-2 with stearic acid (C18:0). The GPI-anchored proteins isolated from the PGAP2 and PGAP3 double-mutant CHO cells had unsaturated acyl chains such as oleic (C18:1), arachidonic (C20:4), and docosatetraenoic (C22:4) acids at the sn-2 position. Recovery of GPI-anchored proteins from the double-mutant cells in the lipid raft fraction is very low, indicating that GPI-anchored proteins become competent to be incorporated into lipid rafts by PGAP2- and PGAP3-mediated lipid remodeling [8]. These observations suggest that the saturated fatty acyl chain of GPI-anchored proteins is required for association with lipid rafts.

Mice deficient in central nervous system GPI (deletion of PIGA in the Nestin-Cre lineage) show progressive decline with severe ataxia consistent with defects in their cerebellar development [9]. Bi-allelic variants in the GPI transamidase subunit PIGK cause a neurodevelopmental syndrome with hypotonia, cerebellar ataxia, cerebellar atrophy, and epilepsy [10]. PIGQ-related GPI deficiency causes nonprogressive congenital ataxia, epilepsy and cerebellar atrophy [11]. Loss of the N-acetylgalactosamine side chain of the GPI-anchor (PGAP4-knockout mice) reduces locomotion activity and accelerates the prion disease pathology [12].

## 4. Single-Molecule Imaging

Suzuki and Kusumi reported the signal transduction mechanism of GPI-anchored proteins via lipid rafts using single-molecule imaging techniques [5]. They found two types of lipid rafts. One is the metastable raft domains with diameters between 2–20 nm, often detected in steady-state quiescent cells, and the other is the stabilized greater raft domains induced by extracellular stimulation and (physiological and artifactual) cross linking of raftophilic molecules. In the plasma membrane of quiescent cells, GPI-anchored protein rafts are dynamic in the sense that they form and break continually. The ligand-induced GPI-anchored protein cluster rafts recruit ganglioside and the Src-family kinases, and trigger downstream signaling [13,14]. The ganglioside GM1, clustered by the addition of the cholera toxin B subunit or GM1 antibody, also recruits the Src-family kinases, suggesting that the transbilayer raft phases induced by the outer-leaflet stabilized rafts recruit lipid-anchored signaling molecules in the inner leaflet by lateral raft–lipid interactions there, and the transbilayer raft phase serves as a key signal transduction platform.

## 5. Gangliosides

**Figure 1:**
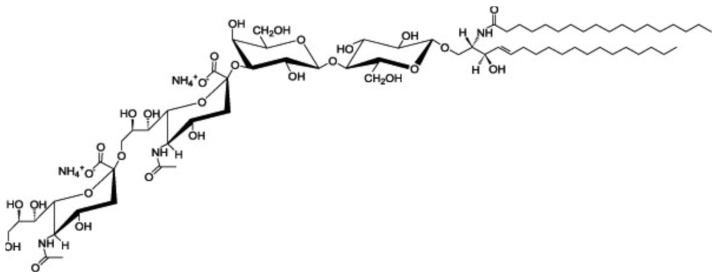
Structure of ganglioside GD3 (cited from https://avantilipids.com/product/860060 (accessed on 9 March 2023)).

Ganglioside GD3 is a major ganglioside in neuronal progenitor cells (Figure 1). Highly sialylated gangliosides, GM1, GD1a, GD1b, GT1b are the main gangliosides in adult neurons. GM2/GD2 synthase knockout (KO) mice, expressing only ganglioside GM3 and GD3, showed mild neurological dysfunction at birth and progressive neurodegenerative changes. In contrast, double KO (DKO) mice, of GM2/GD2 synthase and GD3 synthase, expressing only ganglioside GM3, exhibit severe neurodegeneration with earlier onset and wider pathology distribution. Progressive tremor and staggering gait are observed in the DKO mice, suggesting that neurodegeneration occurred in the cerebellum [15]. GM2/GD2 synthase gene deficiency causes hereditary spastic paraplegia. GM3 synthase deficiency causes severe neurological disorders such as infantile epilepsy, mental retardation, and visual disorders [16]. Interestingly, GPI-anchored proteins and raft markers, caveolin-1 and flotillin-1, dispersed from DRM rafts in the cerebella of DKO mice [15]. These results suggest that gangliosides are involved in the formation of lipid rafts and the maintenance of neurons.

## 6. Ganglioside GD3-Binding Proteins

We have been investigating the biosynthesis of gangliosides during neuronal development, and the association of gangliosides with signal transducers in the central nervous system [6,17,18,19,20,21,22,23,24,25,26,27,28,29,30]. Ganglioside GD3 is important as a precursor of the b and c series ganglioside. We isolated GD3 synthase (α2,8-sialyltransferase) cDNA and found that the GD3 synthase expression was regulated in stage- and spatio-restricted manners in the rat central nervous system [18]. To clarify the function of ganglioside GD3 rafts, we identified ganglioside GD3-binding proteins in the cerebellum by coimmunoprecipitation experiments using an anti-ganglioside GD3 antibody. We demonstrated that anti-ganglioside GD3 monoclonal antibody (clone:R24) coimmunoprecipitates phosphorylated proteins of 40, 53, 56, and 80 kDa from rat cerebellar granule cells.

### 6.1. Heterotrimeric G Protein Goα

The 40 kDa phosphoprotein was identified as the α-subunit of the heterotrimeric G protein Go (Goα) [23]. Goα undergoes translocation to the lipid rafts in the early stage of cerebellar development in an activation-dependent manner. SDF-1α induces the chemoattraction of cerebellar granule cells. SDF-1α is the biological ligand for CXCR4, a G protein-coupled receptor. Treatment with SDF-1α stimulated GTPγS binding to Goα and caused Goα translocation to the DRM fractions and RhoA translocation to the membrane fraction. Chemokine SDF-1α is an attractive guidance cue for tangential migration and a meningeal attractant of granule cells [31]. Immature cerebellar granule cells expressing CXCR4 receptor migrate under the pial surface and meninges in response to the SDF-1α attractive action. Mice lacking either CXCR4 or SDF-1α display abnormal migration of granule cells in the cerebellum [32].

The linkage of Goα to the saturated acyl chains by palmitoylation and myristoylation is considered to facilitate Goα translocation to lipid rafts. The linkage of Gγ to prenyl residues, which contain unsaturated bonds, is considered to facilitate exclusion from the lipid rafts. In cerebellar granule cells, the Goαβγ heterotrimer was also excluded from the lipid rafts. This is probably due to the predominant effect of the Gγ prenyl group over the fatty acids of Goα on the partitioning of the heterotrimer in the rat cerebellum. Therefore, the signal-dependent translocation of Goα to the lipid rafts may be a consequence of the dissociation of the heterotrimer into two components, an α subunit and βγ complex.

### 6.2. Src-Family Kinase Lyn

The 53/56 kDa protein was identified as Src-family kinase Lyn by sequential immunoprecipitation with anti-Lyn antibody [19]. R24 treatment of primary cerebellar cultures induced Lyn activation and rapid tyrosine phosphorylation of an 80 kDa protein. These results suggest the functional association of ganglioside GD3 with Lyn. It is assumed that GD3 crosslinking by R24 treatment leads to coalescence of lipid rafts. This may induce clustering of Lyn and transphosphorylation of tyrosine residue in the kinase domain (activation site Tyr 397: homologous to c-Src Tyr419). Lyn contains a myristoylation site at glycine-2 and a palmitoylation site at cysteine-3.

### 6.3. GPI-Anchored Neuronal Cell Adhesion Molecule TAG-1

We attempted to identify the cell-surface molecules involved in Lyn signaling because Lyn is a nonreceptor-type tyrosine kinase, and we found that R24 coimmunoprecipitates TAG-1, a GPI-anchored neuronal cell adhesion molecule and that the antibody-mediated cross-linking of TAG-1 induced Lyn activation and rapid tyrosine phosphorylation of 80 kDa protein in DRM raft fractions of cerebellar granule cells [21,22].

### 6.4. Csk-Binding Protein Cbp

The 80 kDa phosphoprotein was identified as the Csk (C-terminal Src kinase)-binding protein (Cbp) [25]. Cbp contains palmitoylation sites at cysteine-37, 40. R24 treatment induces tyrosine phosphorylation of Cbp in DRM raft fractions of cerebellar granule cells. These observations suggest that Cbp is a substrate of Lyn. Western blotting with anti-phosphotyrosine antibody demonstrated that the total tyrosine phosphorylation level was higher in the postnatal day 4 developing cerebellum than that in the adult one, and the tyrosine-phosphorylated proteins were highly accumulated in the DRM raft fraction of the postnatal day 4 cerebellum. Lyn protein was present in the DRM raft fraction of both postnatal day 4 and adult cerebella. However, the active form of Lyn was highly accumulated in the DRM raft fraction prepared from the postnatal day 4 cerebellum compared with the DRM raft fraction of the adult one. Tyrosine-phosphorylated 80 kDa protein was immunoprecipitated by the anti-Cbp antibody from the DRM raft fraction prepared from the postnatal day 4 cerebellum. Furthermore, Cbp phosphorylated at tyrosine-314 were highly accumulated in the DRM raft fraction prepared from the postnatal day 4 cerebellum. These findings suggest that TAG-1 transduces intracellular signal at GD3 rafts via Lyn/Cbp during migration of cerebellar granule cells. Taken together, we concluded that GD3 rafts are involved in the migration of granule cells during the early postnatal development. To support this idea, a previous study demonstrated that the lack of GD3 synthase reduces in vitro migration of cerebellar granule cells [33].

## 7. Cerebellar Development

**Figure 2:**
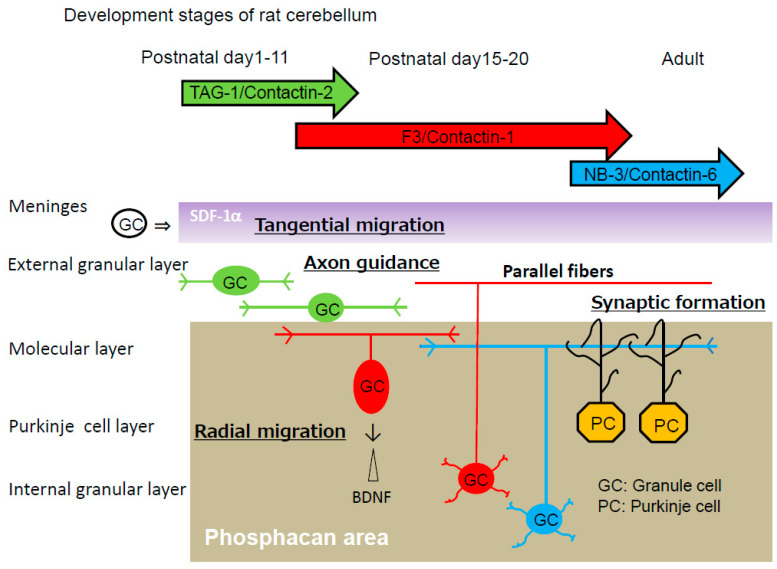
Development of rat cerebellar granule cells. Chemokine SDF-1α (purple), which is expressed by meninges, induces tangential migration of cerebellar granule cells (GC) within the external granular layer. Phosphacan (gray), a chondroitin sulfate proteoglycan, is the repulsive substratum for the adhesion of cerebellar granule cells and promotes the fasciculation of parallel fibers in a parallel manner. Phosphacan also mediates the repulsive effect of radial migration of cerebellar granule cells from the external granular layer. The repulsive effect is because of the interaction of the adhesion molecule TAG-1 with the chondroitin sulfate C of phosphacan. TAG-1, transiently expressed in premigratory granule cells on postnatal days 1–11, prevents radial migration. Downregulation of TAG-1 enables radial migration to the internal granular layer on postnatal day 15. Cerebellar granule cells, via their parallel fibers, form synapses with dendrites of Purkinje cells (PC). Cerebellar granule cells express contactin-2/TAG-1 (green), contactin-1/F3 (red) and contactin-6/NB-3 (blue) in a sequential manner during development.

The cerebellar cortex is organized into four layers during development, including the external granular layer (EGL), molecular layer (ML), Purkinje cell layer (PCL), and internal granular layer (IGL) (Figure 2). Granule cells pass through all the cortical layers of the cerebellum [34]. First, granule progenitor cells migrate tangentially within the EGL, where they differentiate into immature granule cells. Stromal cell-derived factor-1 (SDF-1α), a chemokine expressed by meninges (also known as CXCL12), is an attractive guidance cue for tangential migration and a meningeal attractant of granule cells [35]. Immature cerebellar granule cells expressing the CXCR4 receptor migrate under the pial surface and meninges in response to the SDF-1α attractive guidance cue. SDF-1α prevents radial migration by chemoattracting granule cells toward the pia. Pre-treatment with a raft-disrupting agent, methyl-β-cyclodextrin, inhibits SDF-1α-induced RhoA translocation to the membrane fractions, suggesting that RhoA activation by SDF-1α is dependent on the lipid rafts.

These immature granule cells pause within the premigratory zone of the EGL before migrating through the ML and PCL to the IGL, and then they change their direction by migrating radially along the processes of Bergmann glial cells through the ML. The brain-derived neurotrophic factor (BDNF) appears to be an attractive cue for the migration of cerebellar granule cells. BDNF-expressing cells are restricted to the IGL [36]. The BDNF concentration is higher in the IGL than in the EGL, suggesting that a BDNF concentration gradient could attract granule cells toward the IGL. Accordingly, granule cell migration from the EGL to the IGL is impaired in BDNF-knockout mice [37]. Granule cells detach from glial cells in the PCL and cross the border between the PCL and the IGL. Within the IGL, granule cells migrate radially until they reach their final position at the bottom of the IGL [38]. However, the mechanism by which immature granule cells pause at the EGL/ML interface remains obscure.

**Figure 3:**
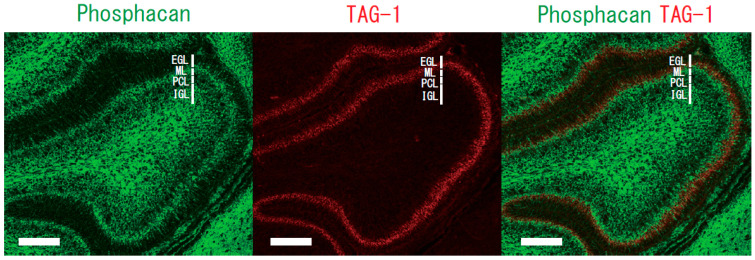
Immunostaining of phosphacan and TAG-1 in sagittal sections from the rat cerebellum. The phosphacan-positive zone in the ML, IGL (green) and the TAG-1-positive zone within the inner part of the EGL (red) of postnatal day 7 rat. EGL, external granular layer; ML, molecular layer; PCL, Purkinje cell layer; and IGL, internal granular layer. Scale bar, 100 μm.

Chondroitin sulfate proteoglycans are major constituents of the extracellular matrix in the brain. Proteoglycans may anchor various attractive and/or repulsive cues, regulating the migration routes of inhibitory neurons [39,40]. Phosphacan, a chondroitin sulfate proteoglycan, is a repulsive cue of cerebellar granule cells [22]. The glycosylphosphatidylinositol-anchored neural adhesion molecule TAG-1 is a binding partner of phosphacan [41], suggesting that the repulsive effect of phosphacan is possibly because of its interaction with TAG-1. The repulsive effect was greatly reduced on the primary cerebellar granule cells of TAG-1-deficient mice [30]. Surface plasmon resonance analysis confirmed the direct interaction of TAG-1 with chondroitin sulfate C. Phosphacan was present in the ML and IGL, but not in the EGL of the rat cerebellum on postnatal days 1, 4, 7, 11, 15, and 20 and in adulthood. In contrast, transient TAG-1 expression was observed exclusively within the inner part of the EGL on postnatal days 1, 4, 7, and 11 (Figure 2). Therefore, TAG-1-expressing cerebellar granule cells may be in contact with phosphacan at the EGL/ML interface (Figure 3). The overlap of the TAG-1 and phosphacan staining, which means that phosphacan and TAG-1 are interacting, is detected in the postnatal day 11 rat. Boyden chamber cell migration assay demonstrated that phosphacan exerted its repulsive effect on the spontaneous and BDNF-induced migration of cerebellar granule cells. These findings suggest that phosphacan may be a barrier-forming molecule that is responsible for the selective repulsion of TAG-1-expressing cerebellar granule cells via GD3 rafts to attenuate BDNF-induced migration signaling. Furthermore, the down-regulation of TAG-1 after postnatal day 11 may enable the switching of granule cells from tangential migration to radial migration to the IGL.

## 8. Contactin Family Members in Developing Granule Cells

The contactin group of immunoglobulin superfamily cell adhesion molecules contains six members: contactin-1/F3, contactin-2/TAG-1, contactin-3/Big-1, contactin-4/Big-2, contactin-5/NB-2 and contactin-6/NB-3. They have been implicated in neural circuit formation. Cerebellar granule cells express contactin-2/TAG-1, contactin-1/F3 and contactin-6/NB-3 in a sequential manner (Figure 2) [42]. 

**Figure 4:**
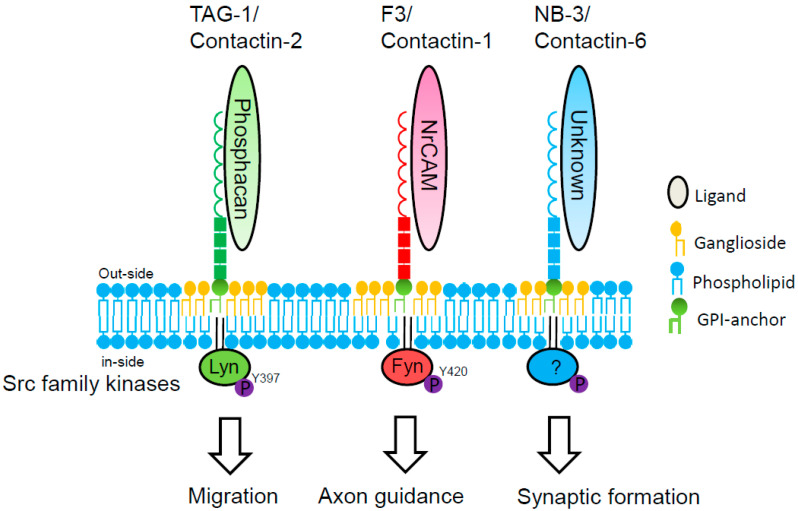
Signal transduction of contactin family members in cerebellar granule cells. Phosphacan binds in trans to contactin-2/TAG-1 on cerebellar granule cells [30]. TAG-1 transduces intracellular signal via Src-family kinase Lyn in lipid rafts of cerebellar granule cells [21]. NrCAM, the L1 family of the immunoglobulin superfamily, binds in trans to contactin-1/F3 on cerebellar granule cells [45]. F3 transduces intracellular signal via Src-family kinase Fyn [46]. Ligand and intracellular signaling of contactin-6/NB-3 in cerebellar granule cells are unknown.

Contactin-2/TAG-1 is expressed by granule cells extending bipolar processes in the inner EGL, and is required for the initial navigation of parallel fibers. Axonin-1, a homolog of chick contactin-2, is also expressed in post-mitotic granule cells as they extend their processes, the parallel fibers. In the absence of axonin-1, these processes still extend, but no longer in a parallel manner, to the pial surface of the chicken cerebellum [43]. Furthermore, ectopic clusters of granule cells are present at the pial surface of the cerebellum of adult TAG-1-null mice, suggesting that these are granule cells that fail to migrate from the EGL [44]. Ligation of contactin-2/TAG-1 with phosphacan induces Lyn activation in lipid rafts of granule cells (Figure 4).

Contactin-1/F3 is expressed by older granule cells in the ML, and required for parallel fiber fasciculation. Contactin-1 mutant mice display a severe ataxic phenotype consistent with defects in the cerebellum [47]. Analysis of the contactin-1 mutant cerebellum reveals defects in granule cell axon guidance and in dendritic projections from granule and Golgi cells. Medaka and zebrafish contactin-1 mutants show abnormal for motor coordination [48]. The contactin-1/F3-associated kinase is identified as Fyn in cerebellum. NrCAM is the functional receptor for F3 mediating the regulation of axonal elongation from cerebellar granule cells [45].

Contactin-6/NB-3 is expressed in granule cells during synaptogenesis with Purkinje cells. Contactin-6/NB-3 KO mice show impairment in motor coordination and synapse formation between parallel fibers and Purkinje cells [49]. Identification of the Src-family kinase involved in contactin-6/NB-3 signaling remains to be explored. Contactin-5/NB-2 is expressed by subpopulations of Purkinje cells and neurons of the deep cerebellar nuclei.

Lipid rafts are spatially and compositionally heterogeneous in the cell membrane. For example, DRM rafts are separated into two distinct subfractions in dorsal root ganglionic neurons. The GPI-anchored prion protein, located primarily in the cell body, was relatively soluble in detergent. Thy-1, abundantly expressed in neurites, was highly resistant to detergent solubilization [50]. In primary cerebellar granule cells obtained on postnatal day 7 and cultured for 7 days, the ganglioside GD3 and contactin-2/TAG-1 preferentially localized in the cell body, whereas the ganglioside GD1b and contactin-6/NB-3 localized in not only the cell body but also neurites. These observations suggest that there is functional heterogeneity between GD3 rafts and GD1b rafts in cerebellar granule cells. Contactin-2/TAG-1 transduces intracellular signals via ganglioside GD3 rafts in cerebellar granule cells, whereas contactin-6/NB-3 might transduce intracellular signals via ganglioside GD1b rafts. 

## 9. Cerebellar Raft-Binding Proteins 

Cerebellar rafts function as dynamic membrane microdomains for the attachment of various proteins such as adhesion molecules, receptors, signaling molecules, adaptor proteins, and effector proteins (Table 1).

### 9.1. Cell Adhesion Molecule L1

L1 is a member of the immunoglobulin superfamily implicated in a variety of processes, including neurite elongation, axon fasciculation, and migration of neuronal precursors. Localization of L1 to lipid rafts is developmentally regulated. In mouse cerebella, L1 is detected in the DRM raft fraction only between postnatal days 3–28, corresponding to the period when granule cells migrate and neurites elongate in vivo. Growth cone migration mediated by L1 is inhibited after DRM disruption by micro-scale chromophore-assisted laser inactivation of GM1 gangliosides [70]. c-Src is a component of the intracellular signaling pathway in L1-mediated axonal growth of cerebellar neurons [71]. Hypoxic-ischemic encephalopathy (HIE) is the brain injury caused by oxygen deprivation to the brain. The neonatal cerebellum is particularly vulnerable to the effects of HIE. Hypoxic-ischemic injury caused significant increases in the percentage of L1 in the lipid rafts of the cerebellum which persisted until 72 h. Lipid rafts may be a new target for interventions of HIE [72]. Fetal alcohol syndrome is a condition in a child that results from alcohol exposure during the mother’s pregnancy. Fetal alcohol syndrome causes brain damage and growth problems. The similarities between children with fetal alcohol syndrome and those with mutations in the gene encoding L1 implicates L1 as a target of ethanol developmental neurotoxicity. Ethanol inhibits the neurite outgrowth promoting function of L1, and downstream signaling of L1 including activation of c-Src and ERK1/2 in cerebellar granule cells. Following treatment of cerebellar granule cells with ethanol, L1 shifts into lipid rafts [73].

### 9.2. Heterotrimeric G Protein Gsα

The heterotrimeric G protein, Gs α-subunit (Gsα) is enriched predominantly in lipid rafts in subjects with a major depressive disorder [74]. Gsα is normally distributed between non-raft regions where it promotes neurotransmitter-activated adenylyl cyclase activity and lipid raft regions where the adenylyl cyclase activation of Gsα is impaired. Antidepressants promote movement of Gαs out of lipid rafts, and enhanced stimulation of adenylyl cyclase promoting increased cAMP production, activation of cAMP-dependent protein kinase, phosphorylation of cAMP response element binding protein, and increase in BDNF transcription and translation. BDNF knockout ablates antidepressant effects in mice. Both humans and mice with the BDNF val66met allele are more vulnerable to stress-induced anxiety and depression. Human postmortem cerebella in depression show increases in Gsα localization in lipid rafts [59] and decreases in BDNF expression [75,76].

### 9.3. L-Type Voltage-Dependent Calcium Channels

Membrane depolarization causes Ca^2+^ influx through L-type voltage-dependent calcium channels (L-VDCC), which promotes the activity-dependent survival of cerebellar granule cells. Primary cerebellar granule cells can be maintained in a medium containing depolarizing levels of KCl (25 mM), a condition that mimics neuronal activity. Cerebellar granule cells undergo apoptosis when cells are transferred from a medium with 25 mM KCl to a 5 mM KCl medium. The activation of L-VDCC prevents cerebellar granule cells from entering low-K^+^-induced apoptosis. L-VDCC, protein kinase A (PKA), and calmodulin-dependent protein kinase II (CaMK-II) are associated with lipid rafts of cerebellar granule cells [53]. Lipid raft-disrupting agent methyl-β-cyclodextrin decreases the phosphorylation level of the L-VDCC β_2_ subunit and the steady-state calcium concentration in neuronal somas ([Ca^2+^]i) to values close to those measured in 5 mM KCl proapoptotic conditions. These observations suggest that lipid rafts play a major role in the control of the PKA- and CaMK-II-induced phosphorylation level of the L-VDCC β_2_ subunit, thus preventing CGNs from entering apoptosis.

## 10. Conclusions

A number of studies have examined the role of lipid rafts in physical properties and signaling. In contrast, the role of gangliosides in lipid rafts remained obscure. Recent studies by gene targeting of ganglioside synthases revealed ganglioside-specific phenotypes. Lipid rafts are compositionally and functionally heterogenous in the neuronal membrane. We demonstrated that ganglioside GD3 rafts are involved in the migration of granule cells during the early stage of cerebellar development. Ganglioside species-specific raft functions in cerebellar health and diseases remain to be explored.

## Figures and Tables

**Table 1:** Cerebellar raft-binding proteins.

Molecules	Function	Tissue/Cell	Ref.
Amyloid β(1–42)	Major plaque component in Alzheimer’s disease	CGC	[51]
α2δ-2	Calcium channel	cerebellum	[52]
CaV2.1	Calcium channel alpha1 subunit	cerebellum	[52]
Calmodulin	Multifunctional calcium-binding messenger protein	CGC	[51]
CaMK-II	Calmodulin-dependent protein kinase II	CGC	[53]
Cbp/PAG	Csk-binding protein	CGC	[25]
Cyb5R	Cytochrome b5 reductase	CGC	[54]
F3/contactin-1	GPI-anchored adhesion molecule	cerebellum	[46]
Flotillin-1	Scaffolding protein	cerebellum	[52]
Flotillin-2	Scaffolding protein	cerebellum	[55]
Fyn	Src-family tyrosine kinase	cerebellum	[46]
GABA A receptor	Ligand-gated ion channel	CGC	[56]
GABA B receptor	G-protein coupled receptor	cerebellum	[57]
Goα	Heterotrimeric G protein	CGC	[23]
GAP-43	Component of the axon and presynaptic terminal	CGC	[58]
Gsα	Heterotrimeric G protein	cerebellum	[59]
GSK-3	Glycogen synthase kinase-3	CGC	[60]
Hsc70	Molecular chaperone	cerebellum	[61]
Hsp90	Molecular chaperone	cerebellum	[61]
Hsp60	Molecular chaperone	cerebellum	[61]
Hsp40	Molecular chaperone	cerebellum	[61]
LRP1	LDL receptor-related protein-1	CGC	[62]
L-VDCC	L-type voltage-dependent calcium channels	CGC	[53]
Lyn	Src-family tyrosine kinase	CGC	[19]
NCAM 120	GPI-anchored adhesion molecule	cerebellum	[46]
NCX	Sodium–calcium exchangers	CGC	[63]
NgR1	GPI-anchored Nogo-66 receptor	CGC	[64]
NMDA receptor	Ionotropic glutamate receptor	CGC	[65]
nNOS	Neuronal nitric oxide synthase	CGC	[65]
P2X3 receptor	Ionotropic purinergic receptor	CGC	[66]
P2Y1 receptor	Metabotropic purinergic receptor	cerebellum	[55]
p75NTR	Low-affinity nerve growth factor receptor	CGC	[64]
PMCA4	Ca^2+^-ATPase calcium pumps	cerebellum	[67]
Protein kinase A	cAMP-dependent protein kinase	CGC	[53]
Protein kinase C	Ca^2+^-activated phospholipid-dependent protein kinase	cerebellum	[68]
PrPC	Prion	CGC	[58]
SBEE	Serine base exchange enzyme	cerebellum	[68]
TAG-1/contactin-2	GPI-anchored adhesion molecule	CGC	[21]
Thy-1	GPI-anchored adhesion molecule	cerebellum	[46]
Tubulin	microtubule	CGC	[69]

## Abbreviations

BDNF	brain-derived neurotrophic factor
CaMK-II	calmodulin-dependent protein kinase II
Cbp	Csk (C-terminal Src kinase)-binding protein
CGC	cerebellar granule cells
DKO	double knockout
DRM	detergent-resistant membrane
EGL	external granular layer
ERK	extracellular signal-regulated kinase
GC	granule cells
Goα	heterotrimeric G protein Go α-subunit
GPI	glycosylphosphatidylinositol
Gsα	heterotrimeric G protein Gs α-subunit
HIE	hypoxic-ischemic encephalopathy
IGL	internal granular layer
KO	knockout
L-VDCC	L-type voltage-dependent calcium channels
ML	molecular layer
PCL	Purkinje cell layer
PGAP	post GPI attachment to proteins
PIG	phosphatidyl inositol Glycan
PKA	protein kinase A
PKC	protein kinase C
SDF-1α	stromal-cell derived factor-1
TAG-1	transient axonal glycoprotein-1
